# “Research Usually Sits on Shelves, Through the Play It Was Shared.” Co-producing Knowledge Through Post-show Discussions of Research-Based Theatre

**DOI:** 10.3389/fsoc.2019.00048

**Published:** 2019-06-19

**Authors:** Gillian Lewando Hundt, Maria Clasina Stuttaford, Claudette Bryanston, Christine Harrison

**Affiliations:** ^1^Warwick Medical School, University of Warwick, Coventry, United Kingdom; ^2^Centre for Health and Social Care Research, Kingston and St. George's University, London, United Kingdom

**Keywords:** research-based Theatre, post-show discussions, co-production of knowledge, widening participation, impact, knowledge translation, public engagement, evaluation

## Abstract

This is a critical analysis of the co-production of knowledge on health care with members of the public attending two research-based plays that were followed by post-show discussions with expert panelists. *Passing On* was developed from the findings of a qualitative research study of family decision making toward the end of life. *Cracked* explored help seeking pathways for young people experiencing psychosis in families of different ethnicities developed from a research study on this topic. The analysis provides critical reflections on the immediate, post-performance impact of research-based Theatre as a strategy to encourage the co-production of knowledge beyond delivery of the performance itself. The plays were developed through partnership working from interview transcripts and joint workshops engaging academics, users and Theatre practitioners (writers, director, actors). Post-show discussions with expert panels were held after each performance to widen participation of the public in the co-production of knowledge to enhance the impact of completed research and stimulate debate. These discussions were recorded and the audience were asked to complete post-show feedback forms. Audience members were researchers, service providers, service users, and carers. This is an analysis of the co-production of knowledge using the feedback forms and transcripts of the post-show discussions. The analysis showed evidence of impact and co-production of knowledge through dialogues that occurred between the audience members, the members of the panel, and the audience and the panel. The discussions covered policy and practice, personal experiences, and Theatre making. The post-show discussions led the public to critically discuss issues with the panel and other audience members thus widening participation in the co-production of knowledge. The feedback forms gave information on the audience demographics and the immediate impact of the performances. Research-based Theatre with post-show discussions and evaluation forms is a strategy for widening participation and engagement with health research findings, through the co-production of knowledge on complex health issues.

## Introduction

This paper explores how post-show panel discussions following research-based Theatre performances are a strategy for the co-production of knowledge during the dissemination phase of health research. The focus of the analysis is the immediate impact of two plays developed from research studies. The play *Passing On* was developed from qualitative research on the experiences of caring toward the end of life. The other play *Cracked* was developed from qualitative research with young people and their families' experiences of help seeking for psychosis. Both plays were written by the playwright Mike Kenny and were 80–90 min in length. The paper explores how audience participation in post-show discussions of research-based Theatre performances furthers the co-production of knowledge about health care. This paper focuses on the immediate impact of the live performances and the post-show discussions. The participatory co-production of knowledge in the development and performance of the plays will be the subject of a subsequent paper.

There has been some debate about the terms knowledge transfer or knowledge translation seeming to imply a straightforward exchange (Greenhalgh and Wierenga, 2011) and recently the co-production of knowledge within health care and research has been used more widely. This term recognizes that the process involves multiple types of knowledge and experience from a plurality of stakeholders and actors (Rycroft-Malone et al., 2016). The term co-production was developed by Hess and Ostrom (2007). She argued for common ownership of public goods and viewed science as a public good. This has been taken up within civic science (Backstrand, 2003), and co-production is viewed within research as providing a space for exploratory interactions between different types of expertise, such as clinical practice, experiential, and authoritative knowledge (Filipe et al., 2017).

A recent study of public involvement in research (National Institute for Health Research, 2015; Staniszewska et al., 2018) recommends fostering co-production which is defined as having six principles (Boyle et al., 2010) of which four apply to the practice of research-based Theatre and post-show discussions. These are: breaking down boundaries, facilitating as well as delivering, promoting mutuality and reciprocity, and recognizing people and their expertise as assets. In developing the plays with clinicians, researchers, service users and Theatre makers, working with service users and having post-show panel discussions with audience members, unexpected dialogues, and interactions occurred that resulted in reciprocal exchanges between lived experience and professional expertise. This paper is an analysis of the post-show panel discussions as a forum for the co-production of knowledge.

### Definition of Research-Based Theatre

Research-based Theatre is situated within the broad area of Applied Theatre, such as that developed by Boal (1979, 2000), by Theatre in Education (O'Toole, 1977), Applied Theatre (Prentki and Preston, 2009), and Ethnodrama (Akroyd and O'Toole, 2010, Davis, 2018). All have a history of stimulating social action. Within health and social care, this is an innovative way to engage stakeholders in the complexities and dilemmas of difficult contested areas. Applied Theatre has also been used to validate research findings (Stuttaford et al., 2006). Research-based Theatre provides a multi-disciplinary platform that enables the impact of original research to extend its reach beyond academic publications and presentations. Experiencing live Theatre performance created from research findings deepens understanding and allows for learning through cognitive and emotional engagement and debate of complex and contested issues during post-show discussions (Lewando Hundt et al., 2010). Research-based Theatre has been found to provide new knowledge and enhance existing knowledge (Colantionio et al., 2008).

Four Theatre genres can be identified from the literature on using Theatre for knowledge transfer/translation in health research: non-theatrical performances, ethnodramas, theatrical research-based performances, and fictional theatrical performances. Non-theatrical performances are conversational or poetic monologs between researchers. Ethnodramas are largely based on the methodology of Augusto Boal and involve data-based vignettes. Theatrical research-based performance “are informed by the research process, but do *not* strictly adhere to the data as script.…this genre may move away from realism and verisimilitude toward the aesthetic and creative power of Theatre as an interpretive, analytic tool” (Rossiter et al., 2008: 136). *Passing On* and *Cracked* fit Rossiter et al.'s (2008) definition. Both productions used some verbatim text from in-depth interviews in primary research studies and engaged with audiences in post-show discussions. The productions also used theatrical devices to stimulate critical engagement of the audience. For example, in *Passing On*, the audience were given queue numbers as they entered the Theatre, positioning them in an Accident and Emergency waiting area, and a life size puppet represented the frail, ill mother. In *Cracked*, poetry inspired by the interview themes was the soundscape for a youth ensemble, with actors as the adult carers attending a support group sitting on chairs placed in a circle.

Evaluation of research-based Theatre is challenging. In post-performance evaluations, there is feedback on both the content and aesthetics. Three main methodologies have been used for evaluating knowledge transfer in Theatre: unstructured feedback, such as reflective journals or informal discussions, structured open-ended questionnaires, and structured quantitative surveys (Rossiter et al., 2008). Here, the data for the post-performance evaluation of both productions consisted of semi-structured feedback forms and audio-recorded post-show discussions between the audience and panelists to capture the nature and dynamics of the co-production of knowledge.

### Ethics

There are ethical issues related to using research-based Theatre (Lafrenière et al., 2012), such as protecting the privacy of research participants and audience members especially in post-show discussions. There was ethical approval given for the anonymized interviews from the primary research studies to be developed into plays to be performed for educational purposes through Chairs' Action of the National Health Service West Midlands Coventry and Warwickshire Ethics Committee that had approved the primary research studies some years previously. The two plays in this paper were developed from anonymized interview transcripts for which the interviewees had given formal written consent. The verbatim text was a composite text from combining different interviews with demographic details altered—i.e., gender, age, situation. The poetry in *Cracked* was inspired by the themes in the qualitative data but did not use the actual words from any of the interviews. For *Passing On*, all interviewees were written to, requesting that they contact us, if they did not wish for their interviews to be included in the development of the play. Two people phoned for further information, but no one requested to be excluded.

For the post-show discussions (two of which were filmed and the remaining 17 audio-recorded or captured with detailed notes) audience members gave their oral consent. Audience members were informed by public announcement prior to the Theatre performances, that post-show discussions would be taking place after a short interval and that they could choose to come back to the auditorium if they wished to take part. They were also informed in the same announcement that a designated health professional was available to answer questions or offer support after the performance. In addition, those that returned were told, that they could request that their comments or contributions be deleted from the recorded material. On average, about 50% of the audience participated in the post-show activities and no-one requested that their contribution be excluded. The size of audiences ranged from 50 to 150 people.

Evaluation forms comprising four questions were on the audience seats together with a summary of the research the play was drawn from. The first two questions were to what extent the play raised awareness and understanding of either decision making toward the end of life (*Passing On*) or about mental health (*Cracked*), and if the post-show discussion did the same. The possible responses were very well, well, not very well, or not at all. The third question invited comments about the performance and discussion. The fourth asked people to specify if they were a health professional, social worker, carer, service user, friend of attendee or performer, regular, or occasional Theatregoer. In this way the evaluation was anonymized and voluntary.

## Methods and Approach

Santé Theatre Warwick (since 2017 Santé Theatre and Media Productions—STAMP) has been a collaboration between academics and Theatre makers seeking innovative ways to enhance the impact of research and encourage public debate (Lewando Hundt et al., 2010) and as a way of validating research findings (Stuttaford et al., 2006). The research-based plays, *Passing On* and *Cracked* both focused on complex health care issues—dying and mental health. These are experiences that affect us all, and in recent years have become part of public debate through campaigns, such as Dying Matters and charities like MIND and Samaritans. The methodology used for developing research-based Theatre provided an opportunity for research participants' voices to be heard in a way that was authentic and that represented them with integrity.

The methodological approach involves several stages. First, published papers and qualitative interviews from a completed research study are read independently and then thematically summarized through discussion with the researchers. The playwright, Mike Kenny (MK) worked dramaturgically with the Theatre director Claudette Bryanston (CB) both in close partnership and in the rehearsal space with the researchers and creative team, to develop drama strategies by subjecting the research data to a performative translation. Students, researchers, and health professionals are involved in developmental drama workshops enabling exploration of knowledge, ideas, issues, and actions.

Rehearsals are interactive with iterative collaboration between the writer, Theatre director, actors, and researchers. To date, live performances have been followed by post-show discussions involving a panel of these co-creators, health, and social care professionals and service providers in debate with the audience. Performances of *Passing On* and *Cracked* took place in Theatre and non-Theatre spaces to audiences that included service users, carers, students, researchers, and health, and social care service providers and the wider public.

### Passing On

Verbatim text from a research study on end of life care that included a medical record review and interviews with bereaved relatives and health professionals (Jackson et al., 2010) were used to create *Passing On*. The Theatre director and co-author (CB) working with the playwright, (MK), and actors with input from the academic researchers and health professionals, developed the play through a series of workshops. In collaboration with Little Angel Theatre a life size puppet representing the dying person was created. The play was developed through a series of workshops with academics and health and social care professionals with the director and writer. A composite verbatim text play was written from the research interviews by the playwright (MK) and revised by the Theatre director (CB) during rehearsals. *Passing On* was performed nine times in 2013 in London and the Midlands with recorded post-show discussions following each performance and filmed excerpts of the play and a post-show discussion can be viewed on line at http://www.stamproductions.co.uk/past-productions/passing-on.

### Cracked

*Cracked* was developed with a similar methodology. The research explored lay understandings of psychosis and patterns of help seeking amongst families of different ethnicities (Islam et al., 2015; Singh et al., 2015). The play used verbatim text derived from interviews with carers of young people who had experienced psychosis. Poetry represented the inner world of the young people and was spoken by a youth ensemble of young people. The play was developed through workshops facilitated by the director (CB), involving Theatre makers (stage designer, writer, poet, and actors), clinical scientists (psychiatrists, clinical ethicist), social scientists (social work academic, anthropologist), service users, and young adults (youth workers, youth ensemble members). The youth ensembles were drawn from different institutions in each touring venue and in total 68 young people attended the rehearsal workshops and took part in the 11 performances. The young people participating were diverse in terms of region, ethnicity, and education. Filmed material of *Cracked* (produced by Zebra Digital) including a 10 min film on psychosis, a 30 min film on the method and process of developing research-based Theatre, and one of a post-show discussion can be viewed on line at www.stamproductions.co.uk/pastproductions/cracked/.

The expert panel members were academic researchers, health and social care professionals, service users, the Theatre director, and cast members. Semi-structured feedback forms were left on the chairs of audience members. In total only 311 audience members completed the feedback forms from *Passing On* and *Cracked* although the two plays were performed to more than 1,000 people. Sixteen post-show discussions were recorded, and transcribed. In three instances, as shown on Table 1 below, there was no audio-recording made but notes were taken. In Theatre settings, there was a short interval at the end of the plays, and roughly 50% of the audience returned for the 13 post-show discussions. There is a lack of information about why people chose not to stay but reasons could have included logistical travel arrangements, domestic commitments or a preference for private reflection. There was no interval after the six performances in non-Theatre settings, so everyone stayed. Information was provided prior to the event as part of the schedule or timetable. The data set consisted of 311 feedback forms and 19 written accounts (transcripts or notes) of post-show discussions. Authors, GH, and MS undertook analysis of the data identifying emergent themes from the transcripts independently. Table 1 summarizes the performance venues of both plays and the different groups of young people participating in *Cracked*.

**Table 1:** Venues of performances of *Passing On* and *Cracked* with young people involved in *Cracked*.

Venues	Passing On	*Cracked*	Youth groups in *Cracked*	Numbers and ages
University of Warwick Lozells Church Sandwell charity	2 1	2 (1 not recorded) 1 (not recorded)	Wolverhampton Theatre Group−4 performances	10 young people 16–18 years old
Nottingham Lakeside Theatre	2	1	B.Tech. students from FE college	16 students 16–18 years old
Derby Theatre		2	University of Derby Theatre interns	6 young people 18–20 years old
Birmingham Repertory Newman College	2	1 1	Drama students−2 performances	15 students 18–20 years old
London Little Angel Theatre, Pub Theatre	2			
Glasgow Platform Theatre		1 (not recorded)	Platform Youth Theatre	9 young people 16–25 years old
Blue Coat School Theatre, Coventry		2	Sixth form students	12 young people 16–18 years old
Total	9 performances	11 performances		68 young people

## Findings

The written and oral comments of audience members are reported here as said or written. As there was no follow up with individuals following the post-show discussions, it was not possible to interrogate their views further. They represent their immediate responses to the event rather than a reflective discussion after a filmed presentation (Adams et al., 2015).

The feedback forms from about 20% of the audience members gave an indication of the audience make-up. They self-identified as students, members of the public who often had personal experiences of the health care situations explored in the play, service users, health care professionals or academics. There were equal numbers of regular and occasional Theatregoers and a third of people identified themselves as never or rarely going to the Theatre. This indicates outreach to non-Theatre goers through using non-Theatre spaces for some performances. For example, *Cracked* was performed as part of the Scottish Mental Health Arts and Film Festival at the Platform-Bridge Theatre in Easterhouse, Glasgow, in a church at a Conference on African Caribbean Men's Mental Health in Birmingham, and at a community center in Sandwell. Both plays were performed to 60 social work students at the University of Warwick in studio space where, for *Passing On*, 22 of whom reported on their forms that they rarely or never went to the Theatre.

The co-production of knowledge was the major emergent theme in the analysis of the post-show discussions and feedback forms and had two sub-themes, (1) the process and impact of Theatre making from research, and (2) participative discussions of issues raised in the plays with a sharing of experiences.

### Co-production of Knowledge Through the Process and Impact of Theatre Making From Research

There were many comments both orally and in writing about the power of Theatre to represent universal experiences that people could respond to cognitively and emotionally knowing that it was developed from the experiences of research participants. Audience members reported how they learnt through the live performance about real life phenomena and were able to generalize from the particular to the more general.

“*Research usually sits on shelves, through the play it was shared. The acting, for me brought out the thoughts/experiences the individual goes through which I had not experienced before”*

(Feedback form, Occupational Therapy Student, *Cracked*, Derby Theatre, 10.10.2015)

“*The play… explores the impact on their carers, the family members, the mums and dads who try to make sense of their child's irrational broken world… We are all a bit cracked, it's part of being human, some more than others.”*

(Feedback form, Charity worker/Regular Theatregoer, *Cracked*, Blue Coat School Theatre, 15/16.10.2015)

Theatre makers on the panel were able to explain how the process of developing research based Theatre and by doing so revealed how the voices of research participants were respected and heard. As the playwright explained:

“*When I started reading the interviews I thought: I can't say that better. I've got to use the true, authentic voices of the people going through these experiences…it's an honest reflection of the experiences that those people went through.”*

(Playwright Mike Kenny, *Cracked*, Derby Theatre, 10.1.2015, afternoon, post-show discussion).

The use of theatrical devices, such as the life-size puppet in *Passing On* were discussed and commented on. Here an actor reflected on the use of the life size puppet to represent her mother.

“*I'm not a very experienced puppeteer…I never at any point think she isn't my real mother, and that's really odd. Because the audiences project onto her, but I think we do as well, and quite often during the show I just sort of think…‘But she does look like my mother!’ Or when she's dying I really believe that she is. And it's a very odd thing when you start to love a puppet.”*

(Actor Ali Belbin, Passing On, Birmingham Repertory Theatre, 7.3.2013 post-show discussion)

Actors in both productions when taking part both in rehearsals and the post-show discussions, expressed how the plays and performances impacted on them and related to their own experiences of bereavement and mental health issues in their own lives. An actor in *Passing On* explained how important it was to respect research participants voices by delivering verbatim text accurately in terms of content and tone. Another actor explained how creating *Cracked* was a dialogue and how everyone contributed their own personal experiences, while keeping the characters being portrayed “real.” By combining personal creativity with the verbatim text, the actor described the process as being “therapeutic.” One member of the cast of *Passing On*, wrote later that the play had sparked more discussion and interest in his family than any production he had taken previously taken part in.

The Theatre student actors responded positively about participating in *Cracked*, both in terms of the opportunity to perform and learn about research-based Theatre, but also in terms of learning about mental health issues:

“*Well obviously for me I did not know a lot about [mental health] because I only know about acting -that is my world. So, getting in contact with this world through what I love is absolutely amazing. Especially for young people, it is really hard to look into something if you're not directly related to it.”*

(Theatre student actor, *Cracked*, Derby, 10.10.2015, afternoon post-show discussion)

Another had learnt about early signs of psychosis from the play:

“*Normally I do role play for medical exams. So it was really interesting to see the early onset and to see all the flags being missed, as it were,…how easy it is to miss, to brush it off…Because obviously you hear a lot in the media about when it's full blown…. but you don't often hear what about the initial symptoms and how terrifying that must be”*

(Theatre student actor, *Cracked*, Derby 10.10.2015, afternoon post-show discussion)

The co-production of knowledge through the process and impact of research-based Theatre was experienced by both actors and audience members. This was further demonstrated in the way that audience members shared experiences during the participative post-show discussions.

## Co-Production of Knowledge Through Participative Discussion and Sharing of Experiences

Audience members expressed that the plays resonated realistically with their own professional or personal experiences. Some health professionals expressed how realistic *Passing On* was concerning caring for someone toward the end of life:

“*Thinking about the work I've done with people with dementia when people lose their sense of speech–the non-verbal becomes much more important. That hand stroking is really significant. Also, when people die that sort of guttural sound of the breath, that is what it sounds like, and it was very realistic.”*

(Audience member, *Passing On*, 22.1.2013, Lakeside Matinee, post-show discussion)

“*I'm a health professional myself and throughout the whole play my friend was saying, I was nodding and shaking. I recognized there the situations that I've come across myself.”*

(Audience member, *Passing On*, 7.2.2013, Little Angel Theatre, London, post-show discussion)

The written feedback of audience members who had experienced mental health issues as service users, showed that they recognized aspects of the play *Cracked* as an “*accurate representation*” (Feedback form User, Lakeside, 30.9.2015), and another expressed that

“*Some of the script, lines and feelings expressed, struck resonance with me and reminded me of times in my life”*

(Feedback form, Service User *Cracked*, Derby Theatre, 10.10.2015)

Some audience members gave written feedback which showed how they related to the situations portrayed in *Cracked* to situations they encountered in their working lives.

“*I work for a Housing Association and deal with complaints from people about their neighbors. I recognized many of the scenarios/issues in the play as issues that are often part of complaints like. My neighbor is keeping me awake by running up and down the stairs all night or My neighbor is staring at me and shouts through the walls at me.”*

(Feedback form, Regular Theatre goer/Friend, *Cracked*, Derby Theatre 10.10.2015)

“*I work within an early intervention service and I felt this was a very powerful portrayal of the experience of psychosis from both the individual and family perspective”*

(Feedback form, Health professional, *Cracked*, Blue Coat School 15/16.10.2015)

Like the actors, some audience members felt that there was a therapeutic element to the plays. One service user felt that seeing *Cracked* felt “*very peaceful and it helped*” (Feedback form, Service user, *Cracked*, Blue Coat School, 15/16.10.2015)

Another service user shared with the audience that:

“*Nobody could fix me because I couldn't fix myself. I was drinking to die at the end of my drinking. Five years on, I'm in a much better place. I work with others and I share my experience honestly and openly because I have no fear today. I'm grateful for this evening because it always helps me to remember to look at me today. So, thank you all.”*

(Audience participant, Derby Theatre, 10.10.2015, post-show discussion)

Another audience member reflected on learning about on help seeking more generally:

“*The play had a deep emotional impact on myself. I could identify with the fact that health authorities don't always understand until a person has reached breaking point. It has also helped to identify certain patterns in mental health.”*

(Feedback form, Experience of mental health issues *Cracked*, Blue Coat School Theatre 15/16.10.2015)

Another sub-theme was how the local as presented in the plays was also global. Audience members from elsewhere wrote on the feedback forms that the narratives as performed in the research-based plays was transferable globally. One student wrote that the situations portrayed in *Passing On* were “*very similar when I compare it with my hometown, Hong Kong”* (Feedback form, 7.2.2013, Little Angel Theatre, London) and another person in the audience at the same performance wrote “*Thoughts between doctors and relatives are the same in my country. It's quite familiar to me.”* (Feedback form, Regular Theatregoer, *Passing On*, 7.2.2013, Little Angel Theatre, London).

Written feedback is not shared with other audience members and so the co-production of knowledge is an individual reflection on the impact of the research-based plays. However, similar comments were expressed and shared in the post-show discussions like this one below.

“*I lost my grandmother a few years ago and I have a medical background so I can relate to the different perspectives as well… I'm coming from different country and I think the perspectives you saw, the family and also the medical ones, are quite international… Even if it's a different system, things happen more or less in similar way.”*

(Audience participant, *Passing On*, Post-show discussion 30.1.2013, Warwick,)

The research-based Theatre performances stimulated discussion about change for example through requests for information and the sharing of experiences and knowledge between service users and service providers. On one occasion an audience member asked “Why do nurses delay responding when a bedpan is needed so that accidents happen after?” Whereupon, a student nurse in the audience, responded that sometimes, she was so busy and fatigued in an understaffed situation toward the end of a shift that it would happen. This is an example of the co-production of knowledge between audience members triggered by the play.

Several audience members described the use of research-based Theatre as thought provoking and authentic and then went on to explain about how the performance and post-show discussion extended their knowledge and understanding. The following three examples of the co-production of knowledge are from student audience members who all felt the performances had extended their understandings of end of life care or help-seeking for psychosis.

“*An excellent production portraying an incredibly realistic story. Beautiful incorporation of puppetry to create a sense of powerlessness and fragility. Learnt a lot about issues surrounding end of life care that I wasn't previously aware of.”*

(Feedback form, Theatre design student, *Passing On*, 22.1.2013, Lakeside, Nottingham)

“*The aim of raising understanding on mental health was definitely achieved – through the research to performance aspect. I think the verbatim and portrayal of psychosis/stories was true*.”

(Feedback form, Drama Theatre Student, *Cracked*, Derby Theatre, 10.10.2015)

“*Amazing, very informative. I am a young person of 15 years old and I think more young people should see this.”*

(Feedback form, feedback form *Cracked*, Blue Coat School Theatre, 15/16.10.2015)

Health and student health professionals expressed how it reminded them about the needs of patients and carers and their skills:

“*The story was very moving - good to have a reminder about thoughts and feelings of relatives and how healthcare professionals' actions have an impact.”*

(Feedback form, Registered nurse (palliative care)/regular Theatre goer, *Passing On*, 22.1.2013, Lakeside, Nottingham)

“*A powerful piece of Theatre, presented in a sensitive manner. Very thought provoking and as a trainee health professional I found it to be a great insight into end of life care and the way in which patient-centered care is essential.”*

(Feedback form, Student physiotherapist, *Passing On*, 7.2.2012, Little Angel Theatre, London)

While the evaluation is limited to immediate impact, there were comments from health professionals who were in the audience showing that the co-production of knowledge was occurring though reflective learning relating to professional practice or behavior:

“*The issues raised in the play and by the post-show discussion were very thought provoking and will make me consider these issues where necessary and ensure that the difficult questions and conversations which are necessary are had in good time before death.”*

(Feedback form, NHS Manager, *Passing On*, 22.1.2013, Lakeside Nottingham)

“*Thought provoking play…made me think about my hospital ward and what we do well or could improve on.”*

(Feedback form, NHS Manager, *Passing On*, 22.1.2013, Lakeside Nottingham)

“*It did make me consider some questions/problems I had not thought about before. It has also encouraged me to want to talk about death more in my personal life and work.”*

(Feedback form, Regular Theatregoer, *Passing On*, 7.2.2013, Little Angel Theatre, London)

The co-production of knowledge was evident in comments from both plays also in relation to the situation of carers as well as the isolation and stigma in these situations.

“*Thank you for the reassurance that mental health is being taken seriously, that the stigma is being taken away and for promoting awareness which will provide support for both sufferers and family that they are NOT alone”*

(Feedback form, Occasional Theatregoer/Carer, *Cracked*, Blue Coat School Theatre, 15/16.10.2015)

It provided insights to people about the experiences of carers:

“*Excellent performance, it really brought home the situation of the position of carers and family and reminded me of when my father died.”*

(Feedback form, *Passing On*, 22.1.2013, Lakeside, Nottingham)

“*The show allowed me to see the good and bad side of caring for people at the last stages of their life. It provided a good glimpse into how people feel and the experiences they go through.”*

(Feedback form, Regular Theatregoer, *Passing On*, 22.1.2013, Lakeside Nottingham)

The research-based Theatre performances stimulated requests for more information during the post-show discussions.

“*What work is actually being done with care homes to change the outcome of, you know, what happened there?”*

(Audience member, *Passing On*, Midlands Art Center, matinee 7.3.2013, Post-show discussion)

“*I'd have welcomed more depth of knowledge on the mental health/psychological side of things i.e., why do people get to the situations in which they find themselves”*

(Feedback form, Occasional Theatregoer/friend/trainee psychotherapist, *Cracked*, Derby, 10.10.2015)

The above two quotes illustrate the importance of the post-show discussion in the engagement process. A member of the panel answered the first question above immediately. The second was written on an anonymous post-show evaluation form with no mechanism for responding.

The combination of performance and post-show discussion was identified by audiences as being important for extending knowledge and understanding.

“*I was more moved by the play's representation of end of life, but together with the post-show discussion issues on end of life care were better understood. They work together to achieve this aim very well.”*

(Feedback form, *Passing On*, 7.2.2013 Little Angel Theatre, London)

We would argue, that the co-production of knowledge on end of life care and psychosis was enhanced by the combination of research-based Theatre performances followed by post-show discussions. The diversity of the panel provided a depth and breadth to the post-performance discussion. The play stimulated people to ask questions and seek information. The discussion was not only a dialogue between different audience members and the panelists but sometimes became a more inclusive discussion including other members of the audience widening both participation and deepening the co-production of knowledge. For example, at performances of *Cracked*, audience members were often keen to ask questions about cannabis use and mental health which then prompted a more general discussion with audience members sharing views and experiences in addition to the responses of the panelists.

Other questions raised by audience members after performances of *Cracked* were about psychosis in relation to gender, UK experiences compared to Europe, links to suicide, and self-harming, trigger points and the influence of social factors. An audience member at the evening performance of *Cracked* in Derby asked about the negative and positive implications of seeking help from religious figures for mental health issues. In responding to this question, academics provided information from the empirical research underpinning the play on this issue, as well as ongoing training interventions with religious leaders, service providers talked about the need for further training of health professionals and another audience member talked about her experiences with religion.

The post-show discussions provided an opportunity to provide information about local services. For example, in the performance of *Passing On* in Sandwell, audience members spoke about the need to prepare emotionally and mentally, not only financially and logistically, for the death of a relative and they also spoke about the need for improved communication between families and health professionals, especially in relation to older people and children. A local resident who also worked as an advocate took the opportunity to signpost people to relevant local services:

*There's information on your chair about compassionate communities and we always advocate it. We hope that bringing the play is part of pushing this with members of the public…. It's opening that discussion with your family and friends*.

(Post-show discussion, Charity Worker, Passing On, Sandwell, 19.2.2013)

Audience members also shared information. For example, a youth worker attending *Cracked* explained how young people feel that social media is sometimes distressing. A panel member at the *Cracked* evening performance in Derby working with young people with mental health issues explained the triggers for psychosis, how it is for young people, carers and health professionals and another responded with the need to continue the conversation started in the post-show discussion after leaving the Theatre. People often stayed on in the Theatre café or bar discussing the play.

The questions in the post-show discussions led to answers from panelists that were an opportunity to signpost audience members to services, publicize where to get further information and communicate evidence and research as well as good practice. In a post-show discussion of *Passing On* (Sandwell 19th February 2013), an audience member observed how the daughter in the play told the doctors she did not want her mother to know she was dying. A panelist responded explaining why health professionals feel they do have a duty to tell patients. At another performance of *Passing On*, local good practice was highlighted during the post-show discussion:

“*I think recently in Nottinghamshire we've seen a really good uptake of training from care home settings and domiciliary care settings as well. Working jointly with the county council we're able to provide a lot of training that we might not be able to do on our own*.

Recently we managed to secure funding through the county council toward the Gold Standards Framework for Care Homes training programme, and we've just found out that one of our homes has got Beacon Status, which is the highest. So things are changing in the care home setting.”

(Trainer, *Passing On*, 22.12.2012, Lakeside evening, Nottingham, post-show discussion)

The shared experiences were practical as well as more general. During the post-show discussions of *Passing On*, people talked a great deal about palliative care options and advance medical directives and two frail elderly members of the audience produced and showed to others, the Do Not Resuscitate cards that they were carrying in their wallets. During the discussions the panelists reported on the impact of the play on others at previous performances:

“*I have a friend who came when, her sister was dying. She said after seeing the play she was able to go and visit her sister and have a conversation about topics that she'd been frightened to talk about, like ‘What do you want the funeral to be like? How do you want to be buried? How can I help you?’ She found the play facilitating.”*

(Academic panelist, *Passing On*, University of Warwick, 27.2.2013, post-show discussion)

“*It can be a trigger can't it? Because the power of Theatre is that you respond to it both intellectually and emotionally so that you can start a different sort of conversation. Definitely a different conversation to reading an academic paper.…So for example people who have seen the play have said to me: I'm going to write a ‘do not resuscitate order’, that is properly dated and is in my home.”*

(Academic panelist, *Passing On*, Little Angel Theatre, London, 7.2.2013).

## Discussion and Conclusions

The emergence of Death and Dying and Mental Health as topics for public debate has been evident in this century. These post-show discussions following the performances of two plays developed from research on death and dying and mental health, respectively, continued the process of public engagement with dialogue and co-production of knowledge on these topics.

The six principles of the co-production of knowledge identified by Boyle et al. (2010) and incorporated into the recent NIHR report (National Institute for Health Research, 2015) are summarized in Box 1.

Box 1 **Six principles of co-production of knowledge.**

1. Building on people's existing capabilities
2. Promoting mutuality and reciprocity
3. Developing peer support networks
4. Breaking down boundaries
5. Facilitating as well as delivering
6. Recognizing people and their experiences as assets. Adapted from Boyle et al. (2010) and NIHR (2016, p.17).

We would argue that post-show discussions following research-based Theatre performances promote the co-production of knowledge in terms of four of these principles (2, 4, 5, and 6). The post-show discussion promoted mutuality and reciprocity between the audiences and the actors and panelists. In this case, there was evidence of boundaries becoming more porous and fluid through the sharing of experiences and expertise and the research being shared beyond academic papers. The post-show discussions facilitated this sharing and the lived experiences of the research respondents and members of the audience were recognized as well as the authoritative knowledge of the panel members. The post-show discussions created an exploratory space and new interactions (Filipe et al., 2017) and were underpinned by the Ostrom's commitment to science as a public good (Hess and Ostrom, 2007).

The panels always included Theatre makers, service users, academics, and health, and social care professionals. They were seated in front of the audience and responded to questions relating to their expertise that the facilitator directed to them. They often elaborated on each other's responses so that there were interactions between both members of the audience and panel members as well as between the panel members. In terms of power dynamics, the tone was informal rather than didactic and the audience led the direction and scope of the discussions. Each one was different in content and reach but all included requests for information, questions on the process of transforming research into Theatre as well as generous sharing of lived experiences.

The audience oral and written responses demonstrated public engagement with research findings and the co-production of knowledge through requests for information, comparing, and contrasting experiences and establishing dialogue with the multi-disciplinary panels (Jones, 2002). The panelists and audiences became collaborators in engaging with the findings of the research and the diversity of the panels was important for connecting with truths from the research and the authenticity of audience experiences (Mitchell et al., 2011). Audience members, Theatre practitioners and panelists connected in new ways and shifted understanding and meaning (Mitchell et al., 2011).

Audience members expressed that they would be more able to talk about sensitive issues, and would be more committed to destigmatizing talking about subjects seldom spoken about freely. The feedback forms showed that audience members felt that the plays conveyed authentic experiences and truths, that through the performances, and the post-show discussions, they had increased their understanding of health care at the end of life and mental health of young people. The post-show discussions had recurring questions and concerns raised by the audience members. These were: How to get help? How to recognize when to seek help? and How to further understandings of the topic? The audience members appreciated that the plays included the perspectives of the service users, carers, and health professionals, and with the use of verbatim text was sensitive and respectful of the participants. Health professionals felt that the plays were authentic representations, true to case histories and life experiences they encountered in their work.

Similarly, carers and users expressed that the portrayal of families' experiences reflected their own. The use of research-based Theatre performances with post-show discussions is a strategy for encouraging extending the co-production of knowledge beyond the performances through enhanced public engagement. The audience members were engaged with the health research and dilemmas in health care represented in the plays and in the post-show discussions with the panelists—academics, Theatre practitioners, health professionals, and users.

There are methodological challenges to capturing the co-production of knowledge and impact of research-based Theatre. The data show the immediate impact of research-based Theatre performances and participative post-show discussions, but as yet there is little evidence of longer-term engagement. Another limitation is that discussions were primarily between the members of the audience and members of the expert panels and to a lesser extent between audience members. Further informal talk continued after the performance and post show discussion that was not captured. In Theatre settings, only about 50% of the audience returned after an interval for the post-show discussion, however, in non-Theatre spaces all the audience remained. In both settings, only a minority actively participated with questions or view but silent participants had chosen to be present. These challenges are a priority to address in future productions as a way of developing methods to capture the multiple layered dimensions of the co-production of knowledge.

Live performances followed by participative discussions reach a limited number of people but has impact, whereas digital versions on line reach far more people. However, we know little about the impact of the on-line material other than the number of likes and dislikes and do not know if it was used by individuals or shown to groups, such as students as part of active learning with facilitated discussion. One of the limitations of this analysis is that it only captures the immediate impact of research-based Theatre. Medium term impact could be measured by contacting audience members 3–6 months after the performance. A more in-depth exploration of the co-production of knowledge could use a pre- and post-evaluation of audience members' understandings before and after performances. Using research-based Theatre with post-show discussions as a strategy for increasing the co-production of knowledge has powerful and immediate impact. To what extent this remains with those participating is still open to question and debate.

## Ethics Statement

This study was carried out in accordance with the recommendations of name of guidelines, name of committee with written informed consent from all subjects. All subjects gave written informed consent in accordance with the Declaration of Helsinki. The protocols were approved by the Birmingham Research Ethics Committee and Coventry and Warwickshire Research Ethics Committee.

## Author Contributions

MS and GL independently identified key themes from the data and jointly drafted the paper. CB and CH reviewed drafts of the paper. All authors were involved in the development of the plays and post show-discussions.

### Conflict of Interest Statement

The authors declare that the research was conducted in the absence of any commercial or financial relationships that could be construed as a potential conflict of interest.
